# FOXM1 maintains fatty acid homoeostasis through the SET7-H3K4me1-FASN axis

**DOI:** 10.1038/s41420-023-01540-9

**Published:** 2023-08-24

**Authors:** Xixi Li, Weijie Su, Honglin Wu, Jiakun Xu, Hongxing Tang, Xiangkun Chen, Zhanqi Yin, Changming Zhang, Jia Yang, Yibing Yang, Nu Zhang, Lixuan Yang

**Affiliations:** 1 Neurosurgery Unit, The First Affiliated Hospital, Sun Yat-sen University, Guangzhou, China; 2 Intensive Care Unit, The First people’s Hospital of Suqian City, Jiangsu Province, Suqian, China; 3 Intensive Care Unit, The Third Affiliated Hospital of Hebei Medical University, Hebei Province, Shijiazhuang, China

**Keywords:** Cancer metabolism, Cancer microenvironment

## Abstract

Reprogramming of metabolic genes and subsequent alterations in metabolic phenotypes occur widely in malignant tumours, including glioblastoma (GBM). FOXM1 is a potent transcription factor that plays an oncogenic role by regulating the expression of many genes. As a SET domain containing protein, SET7 is a protein lysine methyltransferase which monomethylates histone proteins and other proteins. The epigenetic modification of histones regulates gene expressions by epigenetically modifying promoters of DNAs and inter vening in tumor development. Activation of FASN increased de novo fatty acid (FA) synthesis, a hallmark of cancer cells. Here, we report that FOXM1 may directly promote the transcription of SET7 and activate SET7-H3K4me1-FASN axis, which results in the maintenance of de novo FA synthesis.

## Introduction

Glioblastoma (GBM) is a lethal brain malignancy with limited effective medications. Due to its heterogeneous genetic phenotype and complex tumour microenvironment (TME), simple gene-targeted therapies cannot be easily translated into the clinic, and many more biological characteristics of GBM, including metabolism, the TME, etc., need to be recognized and studied.

As a member of the Forkhead Box(Fox) family of transcription factors, Forkhead box M1 (FOXM1), was designated as the 2010 Molecule of the Year for its powerful ability of transcriptional regulation and potent effect on tumorigenesis. FOXM1 protein is comprised of a C-terminal transactivation domain, an N-terminal repressor domain and a forkhead DNA-binding domain. FOXM1 exerts its oncogenic role by transcriptionally regulating the expression of a wide spectrum of downstream genes associated with diverse cellular processes such as energy metabolism, cell cycle, invasion, metastasis, drug resistance, and DNA damage. For example, FOXM1 promotes the proliferation by regulating the expression of cell cycle proteins like CyclinD1, CyclinE2 [1, 2]. FOXM1 also promotes the invasion by regulating the expression of MMP2, MMP9, E-cadherin and so on [3, 4]. FOXM1 also promotes aerobic glycolysis through the direct activation of metabolic genes like IDH1, LDHA, GLUT1 and HK2 [5–7].

Through alterations in gene expression, malignant tumour cells undergo metabolic reprogramming, including increased anaerobic glycolysis and altered glutamine and lipid metabolism, to sustain their rapid proliferation in the oxygen-depleted TME. Lipids are essential for the proliferation of cancer cells because they are the building blocks of cell membranes and function as signalling molecules. As the substrates of lipid synthesis, fatty acids (FAs) are usually taken up exogenously by normal cells but synthesized de novo in cancer cells [8]. Elevated FA synthesis has been indicated to promote tumorigenesis through several pathways and thus may be considered a hallmark of cancer. FAs can be reversibly oxidized to produce energy to meet the demands of growing tumours, and inhibition of FA oxidation suppresses the proliferation of glioma cells [9, 10].

During the synthesis of FAs, the main substrate, acetyl-CoA, is converted by fatty acid synthase (FASN) into palmitate, most of which is then desaturated by stearoyl-CoA desaturase (SCD) to generate monounsaturated FAs (MUFAs) and some of which desaturated by other FA desaturases (FADS) to generate polyunsaturated FAs (PUFAs). FASN is unimportant in normal cells because of their sufficient uptake of dietary FAs [11] but is overexpressed in nearly all epithelial cancers and is closely related to poor prognosis in several cancers, including breast cancer, prostate cancer, bladder cancer, stomach cancer, ovarian cancer, hepatocellular carcinoma and glioma [8, 12–18].

MUFAs are mainly endogenous and protect cells against peroxidation in the presence of reactive oxygen species (ROS), which results in the peroxidation of lipids in the cell membrane. MUFAs have been reported to reduce cell death in GBM [19]. Excess accumulation of ROS and lipid peroxidation products is lethal to cells and initiates a programme of regulated cell death called ferroptosis [20]. Ferroptosis has recently been reported to suppress the malignant phenotype of GBM, inhibiting proliferation, metastasis, angiogenesis and malignant transformation [21, 22]. By increasing the relative MUFA/PUFA ratio, enhanced de novo FA synthesis inhibits lethal membrane peroxidation and protects cells [23].

Here, we demonstrated that elevated FA synthesis promotes self-renewal and suppresses ferroptosis in GBM and investigated a new mechanism involving FOXM1. FOXM1 promotes the synthesis of FAs by regulating the expression of relevant genes at the transcriptional level. Our study suggests that targeting of FOXM1 combined with inhibition of FA synthesis might be a new strategy for the treatment of GBM.

## Results

### FA metabolism dysregulation in GBM

**Fig. 1:**
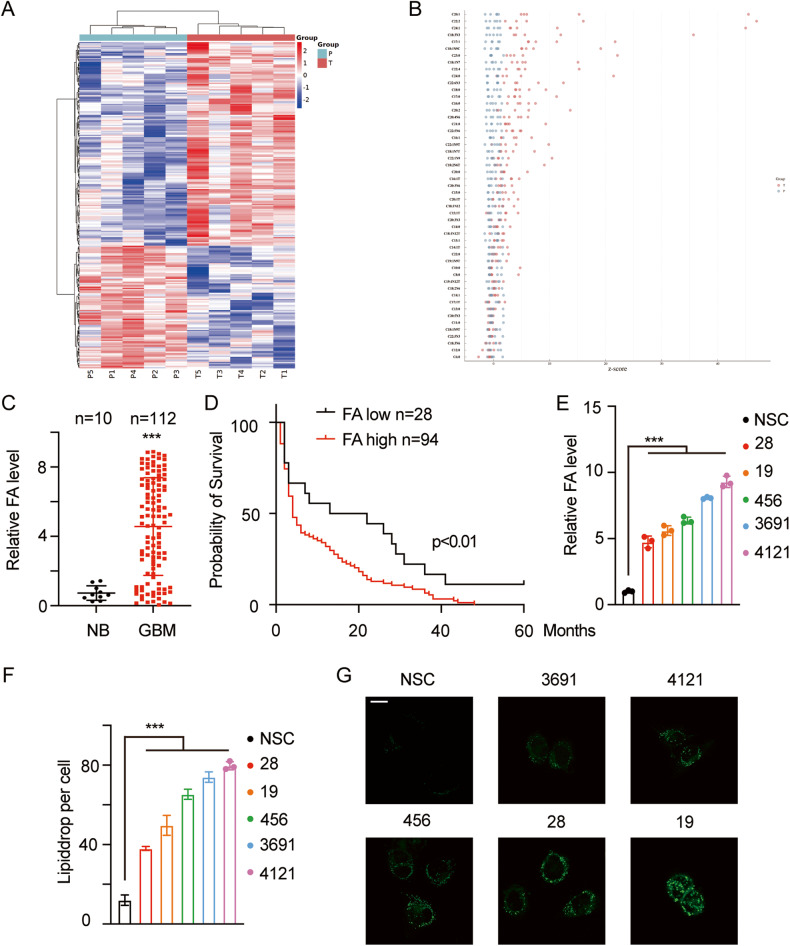
FA metabolism is dysregulated in GBM. **A** A total of 5 GBM and paired normal brain samples were collected and subjected to LC‒MS metabolomic analysis. The DMs were defined as those with *p* < 0.05 and VIP > 1. The heatmap of the DMs is presented (P: paratumoral tissue; T: tumor). **B** *X*-axis: details of the FAs; Y-axis: Z score of each DM; *z* = (x − μ) /σ_o,_ where X is the value of each DM in a certain sample, and μ and σ are the average and the standard deviation in the control group (normal brain tissue). **C** A total of 112 GBM and 10 normal brain samples were collected and subjected to LC‒MS analysis, and the relative FA contents were determined. NB normal brain, GBM glioblastoma; ***p < 0.001. **D** A total of 112 GBM patients were enroled, and the relative FA contents were determined. The patients were divided into two groups according to the FA content. The mean of the whole cohort was considered the cut-off; patients with FA contents higher than the mean were considered the FA-high group, while the other patients were considered the FA-low group. **E** NSCs and 5 patient-derived GSCs were collected and subjected to LC‒MS analysis. The relative FA contents were determined. ***p < 0.001. **F** Lipid droplets were detected using the BODIPY probe, and representative images were acquired by confocal microscopy. The lipid droplets in each cell were quantified (**G**); scale bar, 20 μm. ***p < 0.001.

To generate metabolic profiles of GBM, we collected 5 GBM samples and paired paratumoral normal tissues and subjected them to LC‒MS metabolomic analysis. The metabolites were identified by mass accuracy (<300 ppm), and the MS data were matched with data in HMDB (http://www.hmdb.ca), MassBank (http://www.massbank.jp/), LipidMaps (http://www.lipidmaps.org) mzCloud (https://www.mzcloud.org) and KEGG (http://www.genome.jp/kegg/). The differential metabolites (DMs) were subjected to pathway analysis with MetaboAnalyst and were finally visualized using the KEGG Mapper tool. The DMs were defined as those with *p* < 0.05 and variable importance in projection (VIP) > 1. An orthogonal partial least-squares discriminant analysis (OPLS-DA) plot was generated to verify the quality (SFig. 1A). The heatmap of the metabolites is shown in Fig. 1A. The details of the top DMs are listed in Fig. 1B. Mapping to KEGG pathways identified differential metabolic pathways (SFig. 1B), and FAs attracted our attention among the DMs. The total amount of FAs was significantly higher in GBM samples, and the principal components differed between GBM and normal brain samples (Fig. 1B). We next measured the total FA content in 112 GBM and 10 normal brain samples, and the clinical features of the GBMs are detailed in Supplementary Table 1. FAs accumulated in GBMs and predicted poor outcome (Fig. 1C, D; ***p < 0.001). We next collected neural stem cells (NSCs) and glioma stem cells (GSCs) and subjected them to LC‒MS analysis to measure the FA content. The FA content was higher in GSCs than in NSCs (Fig. 1E; ***p < 0.001). We next detected the amounts of lipid droplets in the different cells using the BODIPY™ FL NHS detection probe. GSCs contained greater amounts of FAs and detected lipid droplets (Fig. 1F, G; ***p < 0.001).

### Identification of the upstream transcription factor by a CRISPR-Cas9 gRNA library screen

**Fig. 2:**
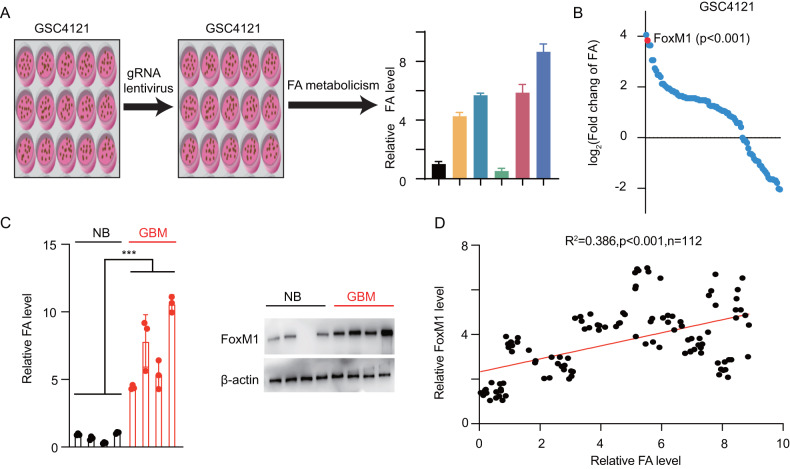
Identification of the upstream transcription factor by a CRISPR-Cas9 gRNA library screen. **A** Schematic showing the gRNA library screening process. **B** Cells transfected with different gRNAs were collected and subjected to LC‒MS analysis to determine relative FA contents. X-axes: gene names, Y-axes: log_2_ fold change values. FOXM1 is indicated. **C** A total of 8 GBM (*n* = 4) and paired normal brain samples (*n* = 4) were collected, FOXM1 expression was determined using an immunoblot assay, and the relative FA content in each sample was determined (uncropped western blots are details in Original Data File). **D** A total of 112 GBM samples were collected, and the relative FOXM1 levels and relative FA contents were determined. Regression analysis was applied to analyse the correlation: *R*^2^ = 0.386, *p* < 0.001.

The specific tumour metabolome is determined by the enzymes in different metabolic processes. Under metabolic stress, specific transcription factors are induced, the downstream gene cluster is activated, and finally, the enzyme is activated or suppressed. To identify potential transcription factors regulating FA metabolism, we transfected GSC 4121 cells with 112 gRNAs to knock out specific transcription factors and measured the total FA content in each cell line (Fig. 2A). The fold change (FC) in each cell line was then calculated, and FOXM1 was finally identified as one of the most important FA metabolism regulators (Fig. 2B). We next collected 4 GBM and paired normal brain samples and subjected them to immunoblotting and LC‒MS to analyse FOXM1 and FAs. GBM samples with higher levels of FOXM1 contained greater amounts of FAs (Fig. 2C; ***p < 0.001). We next determined the relative FOXM1 expression levels and FA contents in a large-scale cohort, and performed regression analysis to detect correlations (Fig. 2D; ***p < 0.001).

### FOXM1-mediated FA metabolism promotes the self-renewal of GSCs

**Fig. 3:**
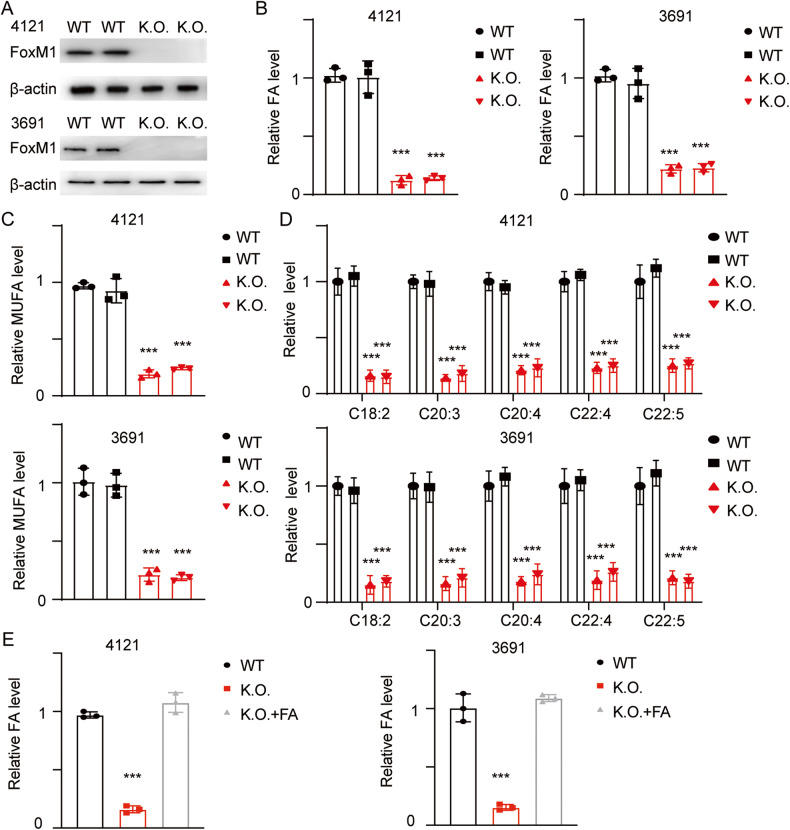
FOXM1-mediated alteration of FA components maintains the self-renewal of GSCs. **A** GSC 4121 and GSC 3691 cells were transfected with gRNAs targeting FOXM1, and FOXM1 was detected using immunoblotting (uncropped western blots are details in Original Data File). **B** The relative FA contents were determined in cells with the indicated modifications. Error bar represent three independent experiments, ***p < 0.001. **C** The relative amounts of PUFAs were determined in cells with the indicated modifications. Error bar represent three independent experiments, ***p < 0.001. **D** The key components of PUFAs were C18:2, linoelaidate; C20:3, eicosatrienoate; C20:4, arachidonate; C22:4, docosatetraenoate; and C22:5, docosapentaenoate. Error bar represent three independent experiments, ***p < 0.001. **E** GSC 4121 and GSC 3691 cells were treated with exogenous FAs, and the relative FA contents were then determined. Error bar represent three independent experiments, ***p < 0.001.

We previously identified FOXM1 as a potential transcription factor regulating FA metabolism. For further verification, we established cell lines with stable knockout of FOXM1 and determined the total amount and the principal components of FAs (Fig. 3A). The total amount of FAs decreased, and the components were altered (Fig. 3B; ***p < 0.001). In FOXM1 knockout (K.O.) cells, the amounts of both PUFAs and total FAs were decreased compared with those in wild-type (WT) cells. The key PUFAs were also evaluated for verification (Fig. 3C, D; ***p < 0.001). We next treated FOXM1 K.O. cells with exogenous FAs. The FA content was completely restored, indicating that the FA uptake ability remained unaffected after FOXM1 knockout (Fig. 3E; ***p < 0.001).

### FOXM1-mediated FA metabolism inhibits ferroptosis

**Fig. 4:**
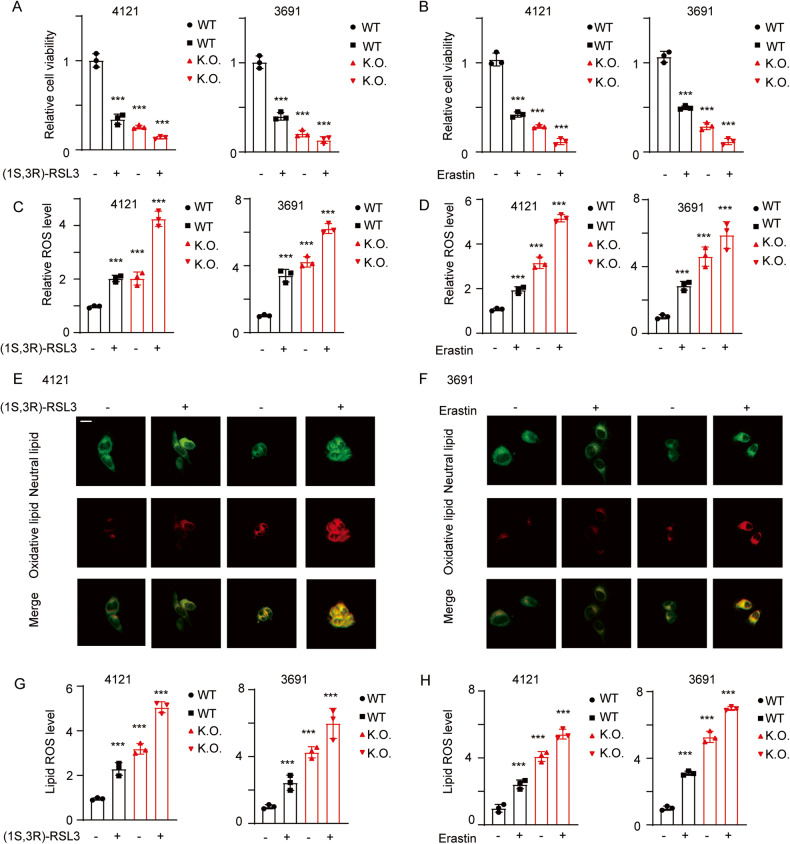
FOXM1-mediated FA metabolism inhibits ferroptosis in GBM cells. **A** Cells were treated with the ferroptosis inducer (1 S,3 R)-RSL3, and the relative cell viability was evaluated. Error bar represent three independent experiments, ***p < 0.001. **B** Viability of each cell line with the indicated modifications. Error bar represent three independent experiments, ***p < 0.001. **C**, **D** The total ROS level was measured in each cell line with the indicated modifications. Error bar represent three independent experiments, ***p < 0.001. **E**, **F** The indicated cells were treated with a ferroptosis inducer and incubated with BODIPY C12 to detect the nonoxidized/oxidized lipids; scale bar, 20 μm. **G**, **H** Lipid ROS levels in cells with the indicated modifications. Error bar represent three independent experiments, ***p < 0.001.

The components of FAs and the MUFA/PUFA ratio have been reported to affect ferroptosis. To verify this observation, we next treated GSCs with the ferroptosis inducers (1 S,3 R)-RSL3 and erastin and determined the cell viability and apoptosis rates. Erastin and (1 S,3 R)-RSL3 impaired the proliferation of GSCs, and in FOXM1 K.O. cells, ferroptosis was significantly enhanced compared with that in WT cells (Fig. 4A, B; ***p < 0.001). We next detected total ROS and lipid ROS levels using BODIPY™ C12. Treatment with erastin and (1 S,3 R)-RSL3 increased the lipid peroxidation, and this effect was enhanced in FOXM1 K.O. cells (Fig. 4C–H; ***p < 0.001).

### FOXM1 promotes de novo FA synthesis through regulation of FASN

**Fig. 5:**
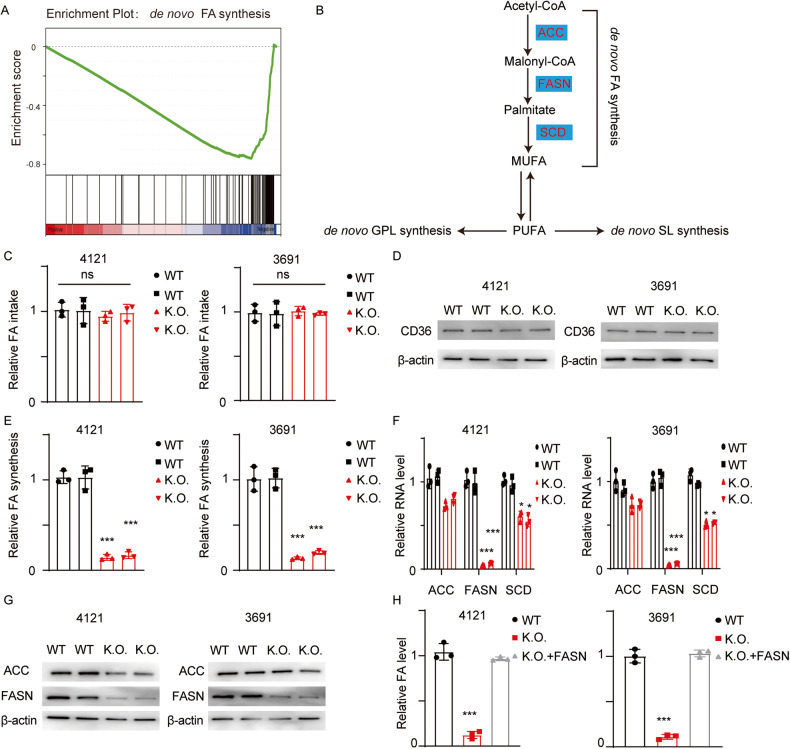
FOXM1 promotes de novo FA synthesis. **A** RNA-seq and GSEA were performed, and the enrichment plot of de novo FA synthesis is presented. **B** The graphical schematic of de novo FA synthesis. **C** The relative FA uptake rate was measured using a FA uptake kit, ns, Error bar represent three independent experiments, not significant. **D** The expression of the key FA transporter CD36 was measured using immunoblotting in cells with the indicated modifications (uncropped western blots are details in Original Data File). **E** Cells were treated with C13-labelled acetic acid, and C13-labelled FAs were then detected. The relative amount of de novo-synthesized FAs was calculated. Error bar represent three independent experiments, ***p < 0.001. **F** The relative RNA levels of the enzymes involved in the de novo synthesis of FA were measured. Error bar represent three independent experiments, ***p < 0.001. **G** ACC and FASN were evaluated using immunoblotting (uncropped western blots are details in Original Data File). **H** FOXM1 K.O. cells were transfected with a FASN overexpression plasmid, and the relative FA content was then measured. Error bar represent three independent experiments, ***, *p* < 0.001.

To further reveal the mechanism by which FOXM1 regulates FA metabolism, we collected cells and subjected them to RNA sequencing (RNA-seq) analysis. KEGG pathway enrichment analysis and gene set enrichment analysis (GSEA) were then performed (Fig. 5A). De novo FA synthesis was significantly dysregulated in FOXM1 K.O. cells. Acetyl-CoA is used to synthesize FAs through a series of enzymatic reactions catalyzed by Acetyl CoA carboxylase (ACC), FASN and SCD (Fig. 5B). Before we examined the change in the de novo FA synthesis pathway, we first determined the FA uptake efficiency using a FA uptake kit and measured the expression of the key FA transporter CD36. The FA uptake efficiency and the expression of CD36 did not differ appreciably between the groups (Fig. 5C, D). We next treated cells with C^13^-labelled acetic acid, which is converted into acetyl-CoA, which is further used for FA synthesis. The FA synthesis efficiency was decreased in FOXM1 K.O. cells; consistent with this finding, the expression of the key enzymes, especially FASN, was decreased, as shown by qRT‒PCR and immunoblotting (Fig. 5E–G; ***p < 0.001). To determine the sufficiency of FASN for FOXM1-mediated FA metabolism, we reexpressed FASN in FOXM1 K.O. cells and measured the total FA content. The FA content was completely restored. (Fig. 5H; ***p < 0.001).

### FOXM1 directly promotes the transcription of SET7

**Fig. 6:**
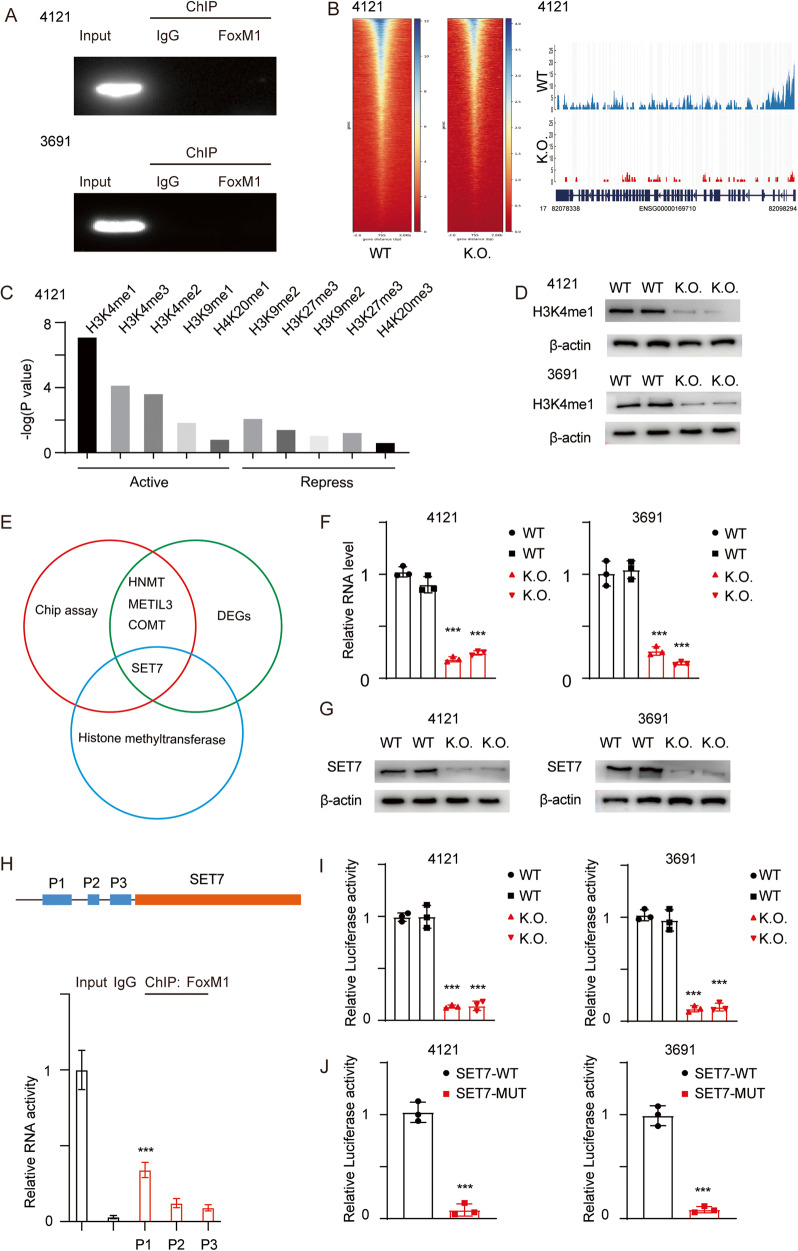
FOXM1 directly promotes the transcription of SET7. **A** A ChIP-PCR assay was performed in GSC 4121 and GSC 3691 cells. **B** Left, heatmap of gene peaks in GSC 4121 cells. Right, FASN peaks in GSC 4121 cells. **C** A Co-IP assay was performed in GSC 4121 cells using anti-H3 and anti-H4 antibodies, and histone methylation was detected using LC‒MS analysis. *Y*-axes: -log_10_ *p*-values. Activating and repressive indicate different functions in epigenetic regulation. **D** H3K4me1 was detected using immunoblotting in GSC 4121 and GSC 3691 cells (uncropped western blots are details in Original Data File). **E** Graphical schematic of the screening strategy and the resulting Venn diagram. **F**, **G** The mRNA and protein levels of SET7 were measured in cells with the indicated modifications (uncropped western blots are details in Original Data File). Error bar represent three independent experiments, ***p < 0.001. ***p < 0.001. **H** Graphical schematic of different primers. Relative mRNA levels were determined using ChIP-PCR (ENCODE SCREEN: P1: EH38E2329942, P2: EH38E2329943, P3: EH38E2329943). **I** The luciferase reporter plasmid was transfected into GSC 4121 and GSC 3691 cells. Relative luciferase activity was determined by calculating the Rluc/Luc ratio. Error bar represent three independent experiments, ***p < 0.001. ***p < 0.001. **J** A SET-MUT reporter plasmid lacking the binding site P1 was constructed. SET-WT and SET-MUT were transfected into GSCs, and relative luciferase activity was measured. Error bar represent three independent experiments, ***p < 0.001. ***p < 0.001.

FOXM1 is a well-known transcription factor that directly regulates the transcription of target genes. We first performed a ChIP assay using an anti-FOXM1 antibody to determine whether FOXM1 directly regulates the transcription of FASN (Fig. 6A). We next hypothesized that the decreased expression of FASN was due to epigenetic repression. We next performed assay for transposase-accessible chromatin and sequencing (ATAC sequencing), and the results indicated that in FOXM1 K.O. cells, the chromatin exhibits a more closed conformation, including at the FASN locus (Fig. 6B). Epigenetic regulation includes two main processes: DNA methylation and histone modification. We next performed a co-IP assay using anti-H3 and anti-H4 antibodies in GSC 4121 cells, followed by quantitative LC‒MS. A summary statistical analysis was performed to assess the multiple functions of each site modification. H3K4 monomethylation (H3K4me1) was identified as the most significantly dysregulated modification (Fig. 6C), indicating that the decrease in FASN expression may be due to the low level of H3K4me1. We also measured the H3K4me1 protein level for verification (Fig. 6D). We next searched the target genes using the following strategy: we downloaded the ChIP sequencing datasets and mapped them to the differentially expressed genes (DEGs) identified by RNA-seq, and we selected histone methyltransferases for further investigation (Fig. 6E). Only SET7 met all the criteria, and we then measured the expression of SET7 using both qRT‒PCR and immunoblotting (Fig. 6F, G; ***p < 0.001). To determine the exact binding site, we designed 3 primers targeting 3 promoter regions upstream of SET7 (ENCODE SCREEN: P1: EH38E2329942, P2: EH38E2329943, P3: EH38E2329943) and performed ChIP-PCR. The results indicated that FOXM1 binds to P1 of SET7 and further promotes its transcription (Fig. 6H; ***p < 0.001). We next transfected a luciferase reporter plasmid into FOXM1 WT and K.O. cells and evaluated the transcriptional activity. Based on our identification of the P1 site, we next established a SET-MUT reporter plasmid without the P1 site and determined the relative luciferase activity (Fig. 6I, J; ***p < 0.001).

### SET7 and H3K4me1 decrease DNA methylation of the FASN promoter

**Fig. 7:**
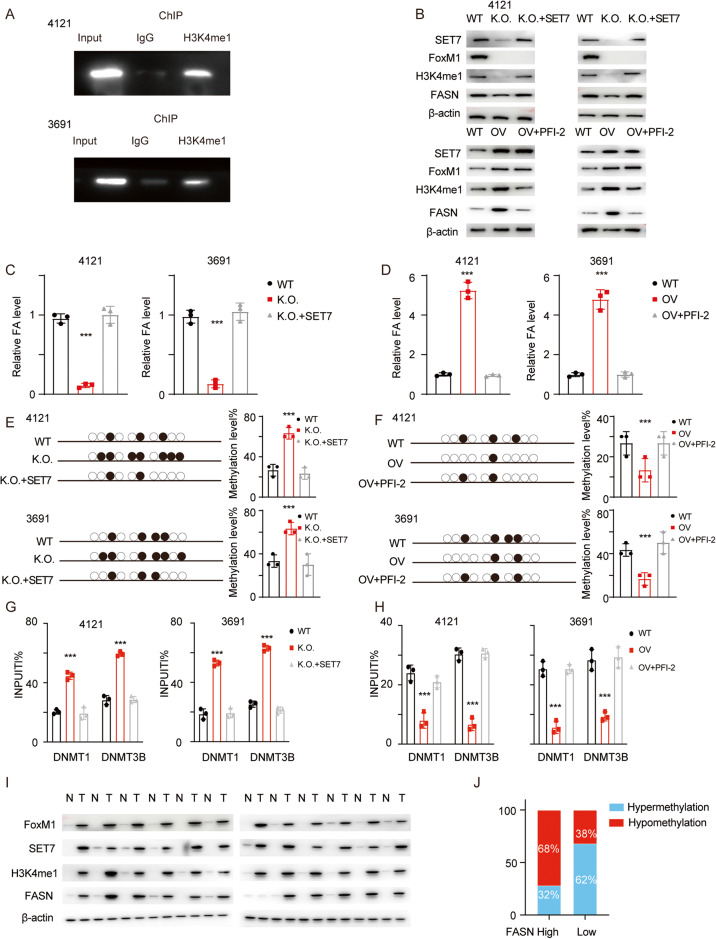
The FOXM1-SET7-H3K4me1 axis maintains FASN expression. **A** ChIP-PCR was performed using an anti-H3K4me1 antibody to detect the FASN promoter. **B** FOXM1 K.O. cells were transfected with a SET7 overexpression plasmid, and FOXM1 OV cells were treated with the SET7-specific inhibitor PFI-2. The levels of FOXM1, SET7, H3K4me1 and FASN were determined using immunoblotting (uncropped western blots are details in Original Data File). **C**, **D** The relative FA content was measured in cells with the indicated modifications. Error bar represent three independent experiments, ***p < 0.001. **E**, **F** BSP was performed to determine the FASN promoter methylation level. A representative image and the statistical analysis results are presented. Error bar represent three independent experiments, ***p < 0.001. **G**, **H** ChIP was performed to detect the involvement of DNMT1/DNMT3B. Error bar represent three independent experiments, ***p < 0.001. **I** The levels of FOXM1, SET7, H3K4me1 and FASN in GBM samples were determined using immunoblotting (uncropped western blots are details in Original Data File). **J** The relative FASN level was determined using qPCR, BSP was then performed to determine the FASN promoter methylation rate, and the proportion of hypermethylation/hypomethylation was calculated. ***p < 0.001.

We next performed ChIP-qPCR to detect the FASN promoter using an anti-H3K4me1 antibody for verification (Fig. 7A). To determine the functional importance of SET7 to H3K4me1 and FASN expression, we reexpressed SET7 in FOXM1 K.O. cells and treated cells with exogenous expression of FOXM1 with the SET7-specific inhibitor PFI-2. H3K4me1 and SET7 were then evaluated using immunoblotting, and the FA content was measured (Fig. 7B–D; ***p < 0.001). We next performed bisulfite sequencing PCR (BSP). The representative methylation levels and statistical analysis are shown in (Fig. 7E, F (***p < 0.001)). As DNA methylation is mediated mainly through DNMTs, we next examined the involvement of different DNMTs using a ChIP assay. In FOXM1-overexpressing (OV) cells, more DNMT1/3B was recruited to the promoter region of FASN (Fig. 7G, H; ***p < 0.001). We next collected GBM samples and evaluated the protein levels of FOXM1, SET7, H3K4me1 and FASN (Fig. 7I). We also determined the DNA methylation levels in the GBM samples and found that the promoter of FASN was hypermethylated in FASN-low samples but hypomethylated in FASN-high samples (Fig. 7J). This pattern was consistent with the expression patterns of SET7 and H3K4me1.

## Discussion

Fatty acid metabolism in tumor has been intensively explored in recent years. It is now clear that dysregulation of FAs synthesis plays important roles in the initiation, progression and drug resistance in many tumors. Glioma cells reprogram their lipid metabolism pathway to synthesize FAs de novo for energy production, membrane synthesis and signalling molecule synthesis. Elevated synthesis of FAs increases the MUFA/PUFA ratio and thus inhibits lethal membrane peroxidation and protects cells [23]. Previous studies reported that overexpression of FASN in glioma correlated with higher WHO tumour grade and poorer prognosis [18, 24, 25]. Targeting FASN induces apoptosis and autophagy in glioma cells and decreases the expression of stemness markers in GSCs [24, 26]. Nevertheless, the molecular mechanism leading to the difference of FA metabolism in cancers from normal tissues still needs to be further illustrated.

In this study, we showed that the FA synthesis pathway was significantly upregulated in GBM, and the greater amounts of FAs in GBM tissue and GSCs further verified the activation of this pathway, consistent with previous studies. A subsequent gRNA library screen for upstream transcription factors revealed that the expression of FOXM1 was correlated with the level of FAs, and the LC‒MS followed by immunoblotting in GBM samples and FOXM1-KO GSCs ultimately confirmed this correlation. Functional experiments in FOXM1-KO GSCs and mice suggested that FOXM1 could promote stemness and inhibit ferroptosis through mediation of FA metabolism. RNA-seq, PCR and immunoblotting results find out FOXM1 regulates FA synthesis through FASN. Screening out ChIP assay, RNA-seq and co-IP assay datasets, SET7 was selected as the downstream regulation molecular of FOXM1. FOXM1 promotes SET7 transcription, and then promotes histone modification in FASN locus, finally causing the FAs metabolism enhancement.

FOXM1 is one of the most frequently overexpressed proteins in human solid cancers. Several studies have found that FOXM1 level is upregulated in GSCs by ALKBH5, SATB2 and other molecular to keep glioma stemness [27, 28]. Few studies have reported the relationship between ferroptosis and FOXM1, and our study revealed that FOXM1 inhibits ferroptosis by increasing FA synthesis, reducing the accumulation of ROS and lipid peroxidation products in glioma cells.

As the 2010 Molecule of the Year, FOXM1 was considered a promising target for cancer therapy, yet the results of few studies have been clinically translated due to the insufficient understanding of the role of FOXM1 in tumours. Increasing evidence has shown that FOXM1 plays a central role in FA metabolism, and Fan et al. identified a set of lipid metabolism genes, including FASN, that might be regulated by the FOXO3-FOXM1 axis [29, 30]. However, few studies have revealed how FOXM1 mediates the dysregulation of FA synthesis. Though FOXM1 is a transcription factor, FASN locus was not found in our ChIP sequencing dataset, and our experimental results indicated that FOXM1 regulated FASN epigenetically via SET7.

The specific mechanism by which FOXM1 reprograms the synthesis of FAs has not yet been reported. The present study showed a synergistic relationship among the expression of FOXM1, FA synthesis, and the expression of FASN, whose sufficiency was then evaluated. Collectively, our findings suggest that FOXM1 regulates FA synthesis through FASN.

## Methods

### LC‒MS metabolite analysis

Metabolites were identified by mass accuracy (<30 ppm) and MS/MS data, which were matched with data in HMDB (http://www.hmdb.ca),MassBank (http://www.massbank.jp/), LipidMaps (http://www.lipidmaps.org), mzCloud (https://www.mzcloud.org) and KEGG (http://www.genome.jp/kegg/).

Ropls software was used for all multivariate data analyses and modelling. After scaling the data, models were built by principal component analysis (PCA), partial least-squares discriminant analysis (PLS-DA) and OPLS-DA.

Metabolic profiles can be visualized as score plots, where each point represents a sample. OPLS-DA allows the identification of discriminating metabolites using the VIP. The *p*-value, VIP value determined by OPLS-DA, and fold change value were applied to discover the contributing variables for classification. Finally, metabolites with a *p*-value of <0.05 and VIP value of >1 were considered statistically significant metabolites. The DMs were subjected to pathway analysis with MetaboAnalyst, which combines the results from powerful pathway enrichment analysis with pathway topology analysis. The metabolites identified by metabolomic analysis were then mapped to KEGG pathways for biological interpretation of higher-level systemic functions. The metabolites and corresponding pathways were visualized using the KEGG Mapper tool.

### GBM samples

Human GBM tumour tissues were obtained from the surgical suite in the Department of Neurosurgery, The 1st Affiliated Hospital of Sun Yat-sen University, after confirmation by board-certified neuropathologists. Tissues were obtained after patients provided informed written consent under a protocol approved by the institution’s Institutional Review Board.

### Cell lines and cell culture

GSC 456, GSC 4121, and GSC 3691 cells were gifts from Dr Jeremy. N. Rich at the University of California, San Diego, CA, USA. The GSC 19 and GSC 23 cell lines were generated through standard procedures. GSCs were cultured as glioma tumour spheres in DMEM/F12 medium supplemented with B27, bFGF and EGF (20 ng/ml each). hNSCs (primary) were obtained from Gibco (TM A15654) and cultured with StemPro® NSC SFM (cat. no. A10509-01) supplemented with 2 mM GlutaMAX™-I Supplement (cat. no. 35050), 6 U/ml heparin (Sigma, cat. no. H3149), and 200 μM ascorbic acid (Sigma, cat. no. A8960).

### Lipid droplets staining and quantification

The staining and quantification of lipid droplets was performed according to the protocol of BODIPY™ FL NHS(D2184). The reaction reagent was dissolved into DMSO at 10 mg/mL, and 1.5 M hydroxylamine with PH 8.5 was used as stop reagent. Cells diluted into the same concentration were rinsed with PBS, and then fixed in 4% Paraformaldehyde (PFA) for 30 min. Cells were then dyed with reaction reagent for 1 h and then stop reagent was added. Confocal microscope was then utilized for observation and quantification of lipid droplets.

### Limiting dilution assay (LDA)

An in vitro LDA was performed according to the LDA protocol. In brief, GSCs and NSCs were seeded into 96-well plates at a different densities (cells per well), and the frequency of glioma spheres in each well was determined. The glioma sphere formation frequency was calculated using ELDA software (http://bioinf.wehi.edu.au/software/elda/).

### Animal study

Athymic (Ncr nu/nu) mice at 6–8 weeks of age were purchased from Nanjing University Farms. Five mice were grouped in each cage. All animal experiments were conducted under Institutional Animal Care and Use Committee (IACUC)-approved protocols at Sun Yat-sen University in accordance with US NIH and institutional guidelines.

### Apoptosis assay

Cells were treated with a ferroptosis inducer and subjected to apoptosis assays following the manufacturer’s instructions for the assay kit (Invitrogen™V35113), which detects the externalization of phosphatidylserine in apoptotic cells by flow cytometry using recombinant annexin V conjugated to red laser-excited allophycocyanin and detects dead cells using a green nucleic acid stain. In brief, apoptotic cells are detected by annexin V binding to externalized phosphatidylserine, and late apoptotic and necrotic cells have compromised membranes that allow the green stain access to cellular nucleic acids.

### RNA-seq and GSEA

Total RNA was extracted using a TRIzol reagent kit (Invitrogen, Carlsbad, CA, USA) according to the manufacturer’s protocol. After digestion with RNase R, RNA was purified with a RNeasy MinElute Cleanup Kit (Qiagen, Venlo, Netherlands). After removing rRNA, RNA was reverse transcribed into cDNA with random primers. Next, the cDNA fragments were purified with VAHTSTM DNA Clean Beads, end repaired, poly(A) tailed, and ligated to Illumina sequencing adapters. GSEA was performed by mapping the DEGs to KEGG pathways and GO terms.

### ATAC sequencing

The ATAC-seq was performed following a standard protocol. Briefly, cells were collected at the same time points and conditions as for the RNA-seq datasets, washed with PBS and treated with accutase. Cells (*n* = 1 × 106) were used to perform the ATAC-seq libraries according to manufacturer’s instructions (Nextera DNA sample preparation kit, Illumina) and size selection (200–800 bp) was performed using Ampure XP beads (Beckman) before next-generation sequencing.

### Quantitative LC‒MS

Total protein was collected and separated by 12% SDS–PAGE, and the band at ~25 kDa was excised and digested. The resulting peptides were analysed by a QExactive mass spectrometer coupled to a nano-LC system (AdvanceLC, Michrom Inc.) The acquired spectra were analysed with the SEQUEST HT algorithm. The methylation levels at different sites were quantified.

### Luciferase activity reporter system

The Renilla luciferase (Rluc) and firefly luciferase (Luc) sequences were amplified from the psiCheck 2 vector (Promega, USA). The Rluc sequence was inserted upstream of the promoter sequence, and Luc was inserted downstream of the promoter sequence. The promoter sequence, along with its 3′ UTR, was amplified and inserted between the Rluc and Luc sequences. Relative activity was determined by normalization to Rluc activity and the activity in control cells.

### qRT‒PCR

Total RNA was extracted with a PureLink RNA Mini Kit (Thermo Fisher Scientific). After reverse transcription, cDNA was purified and subjected to real-time PCR with SYBR Select Master Mix (Thermo Fisher Scientific) in a StepOne Plus real-time PCR system (Applied Biosystems). Target mRNA expression in each sample was normalized to β-actin mRNA expression.

### Bisulfite sequencing PCR

We extracted genomic DNA and accomplished bisulfite conversion from 4121 and 3691 GSC cell lines using EpiTect Fast DNA Bisulfite Kit (QIAGEN). FASN BSP primers were designed for PCR amplification. Then, the PCR products were purified and cloned into pTG19-T Vector. We chose ten subclones from each cell line with three replicated experiments for further sequencing. The number of CpG methylated loci/detected CpG loci was calculated as the methylation level.

### Immunoblot analysis

Equal amounts of protein were loaded into each well of 12% SDS–PAGE gels. After separation and transfer to membranes, the membranes were blocked with 5% BSA and incubated with the corresponding primary antibody at 4 °C overnight. After incubation with the secondary antibody, the bands were visualized with an ECL kit.

### Supplementary information

SUPPLEMENTAL Table 1

SUPPLEMENTAL Figure 1

Original Data File

## Data Availability

The raw data can be provided by corresponding author on reasonable request.
