# Supplementary material for: FOXM1 maintains fatty acid homoeostasis through the SET7-H3K4me1-FASN axis
Source: Cell Death Discov. 2023 Aug 24;9:310. doi: 10.1038/s41420-023-01540-9 (PMC10449838; doi:10.1038/s41420-023-01540-9)
Supplement: Supplementary file 1 — SUPPLEMENTAL Table 1 [file 41420_2023_1540_MOESM1_ESM.docx]

**Table 1**. Clinical features of the 112 glioblastomas patients.

| **Clinical features** | **Total (n= 112)** | **Fatty acid expression** | | ***P*** |
| --- | --- | --- | --- | --- |
|  |  | **high (n= 94)** | **low (n= 28)** |  |
| **Sex** |  |  |  |  |
| Male | 69 | 57 | 12 | 0.793 |
| Female | 43 | 37 | 6 |  |
| **Age (years)** | 49.70 ± 1.18 | 48.93 ± 1.30 | 53.72 ± 2.68 | 0.137 |
| **Maximum tumor diameter (cm)** | 4.84 ± 0.13 | 4.91 ± 0.15 | 4.44 ± 0.33 | 0.200 |
| **Relative FOXM1 level** | 3.66 ± 0.15 | 3.80 ± 0.16 | 2.91 ± 0.32 | 0.025 |
| **Survival time (months)** | 12.21 ± 1.30 | 10.56 ± 1.23 | 20.83 ± 4.58 | 0.003 |
