# Supplementary figures and images for: FOXM1 maintains fatty acid homoeostasis through the SET7-H3K4me1-FASN axis
Source: Cell Death Discov. 2023 Aug 24;9:310. doi: 10.1038/s41420-023-01540-9 (PMC10449838; doi:10.1038/s41420-023-01540-9)

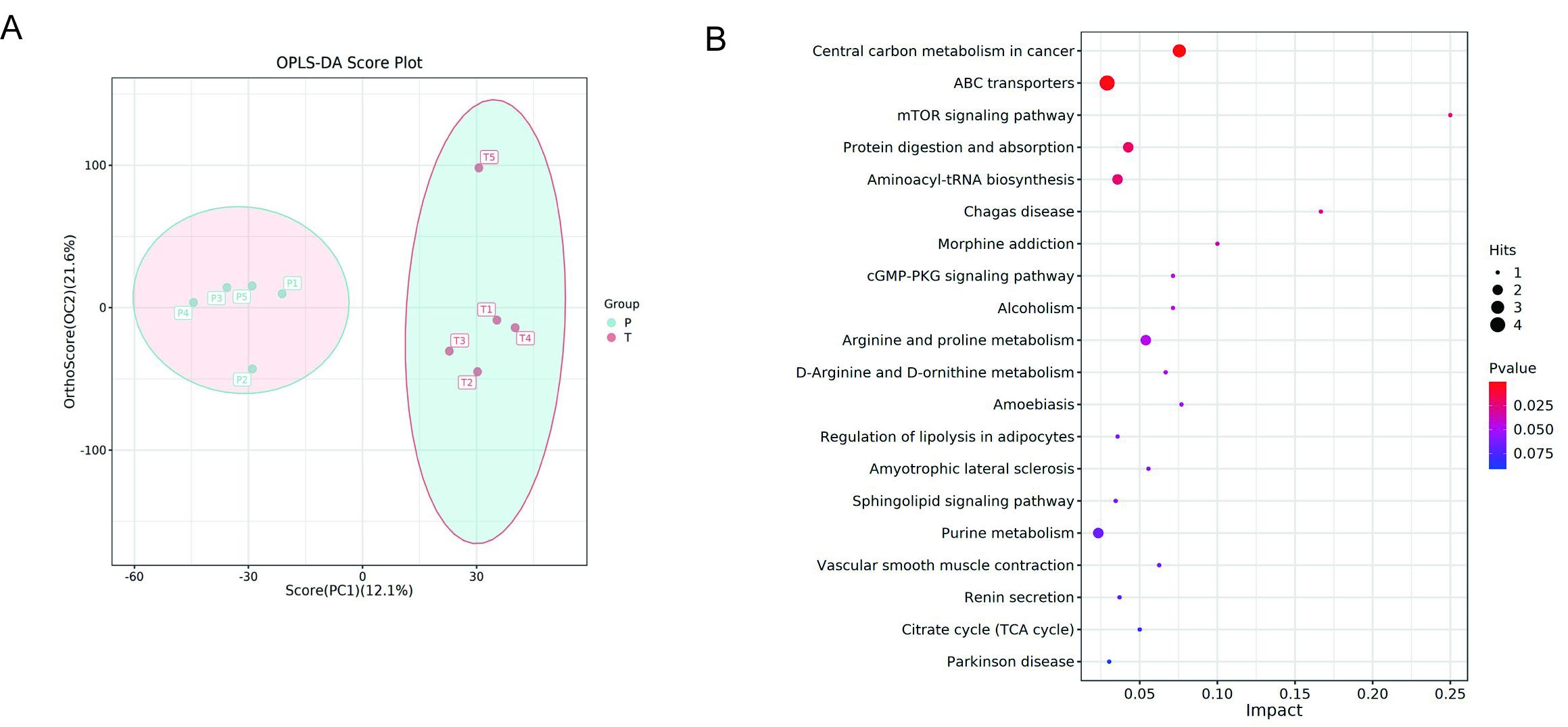

Supplement: Supplementary file 2 — SUPPLEMENTAL Figure 1 [file 41420_2023_1540_MOESM2_ESM.tif]

# Figure2

2C

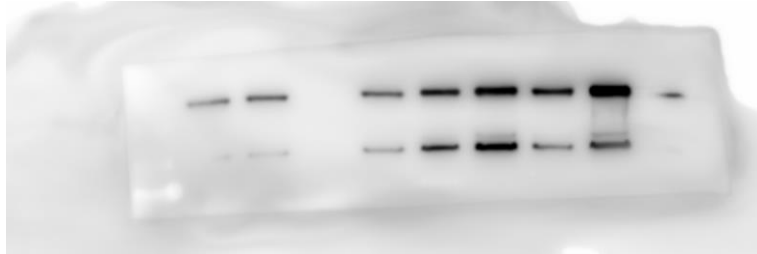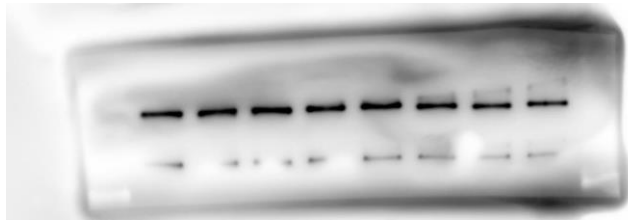

# Figure3

3A

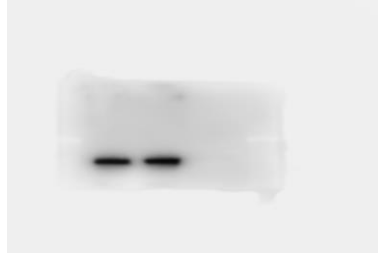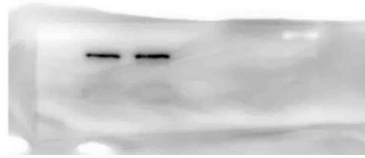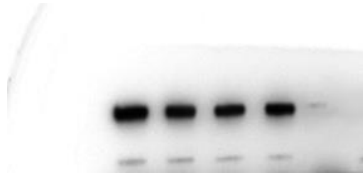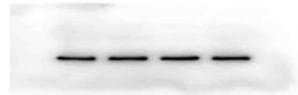

# Figure5

5D

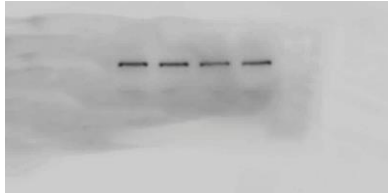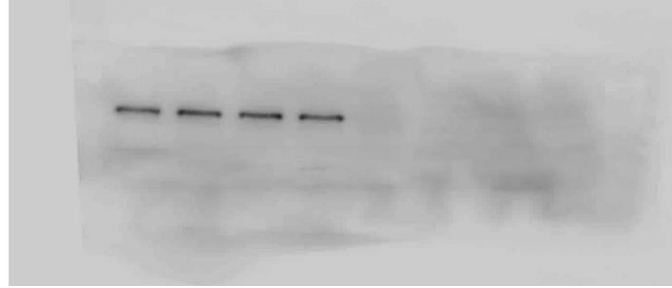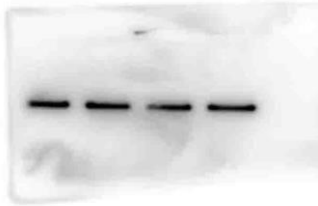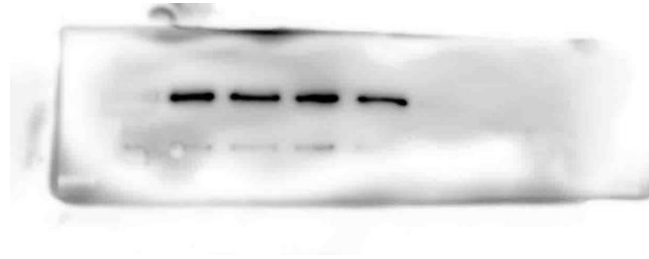

5G

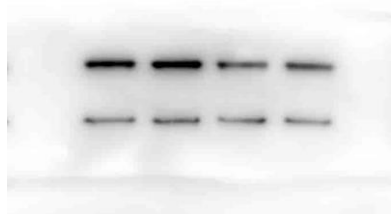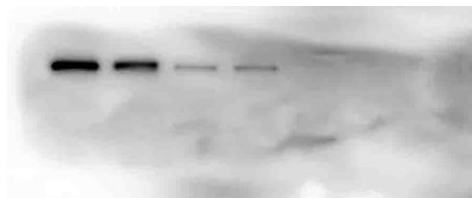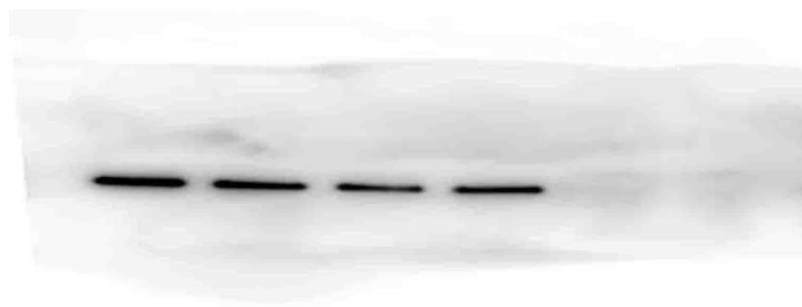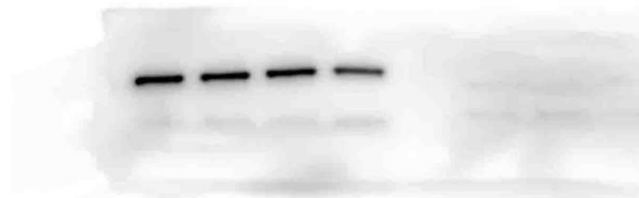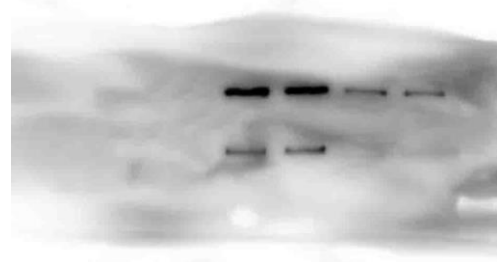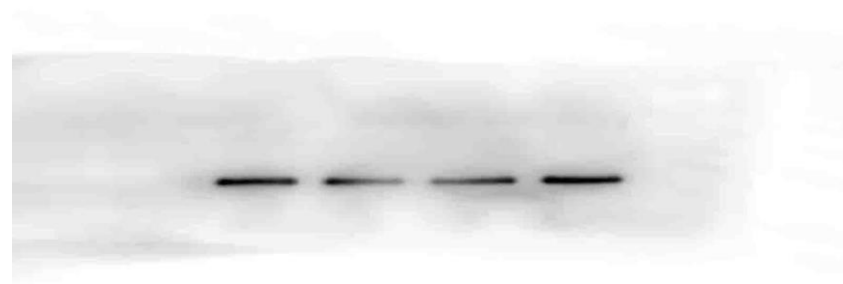

# Figure6

6D

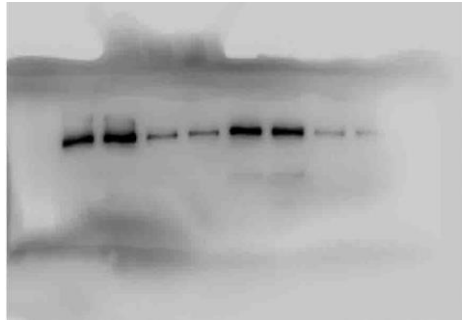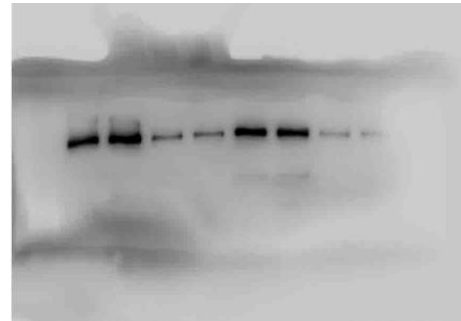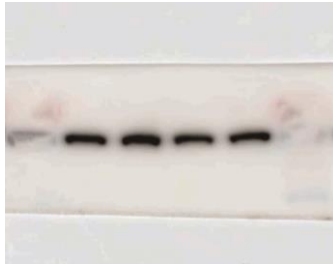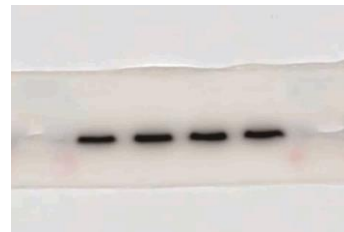

6G

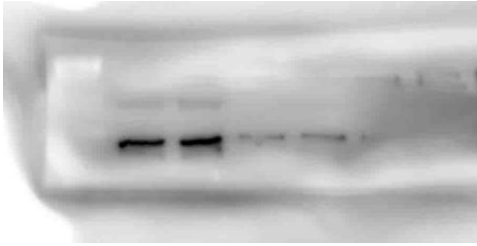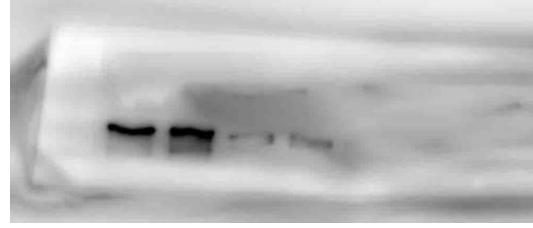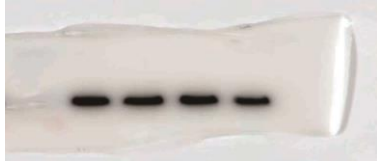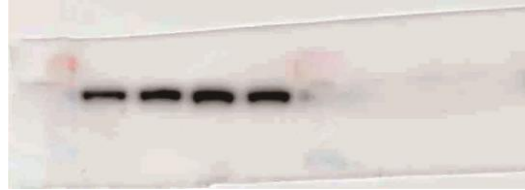

# Figure7

7B

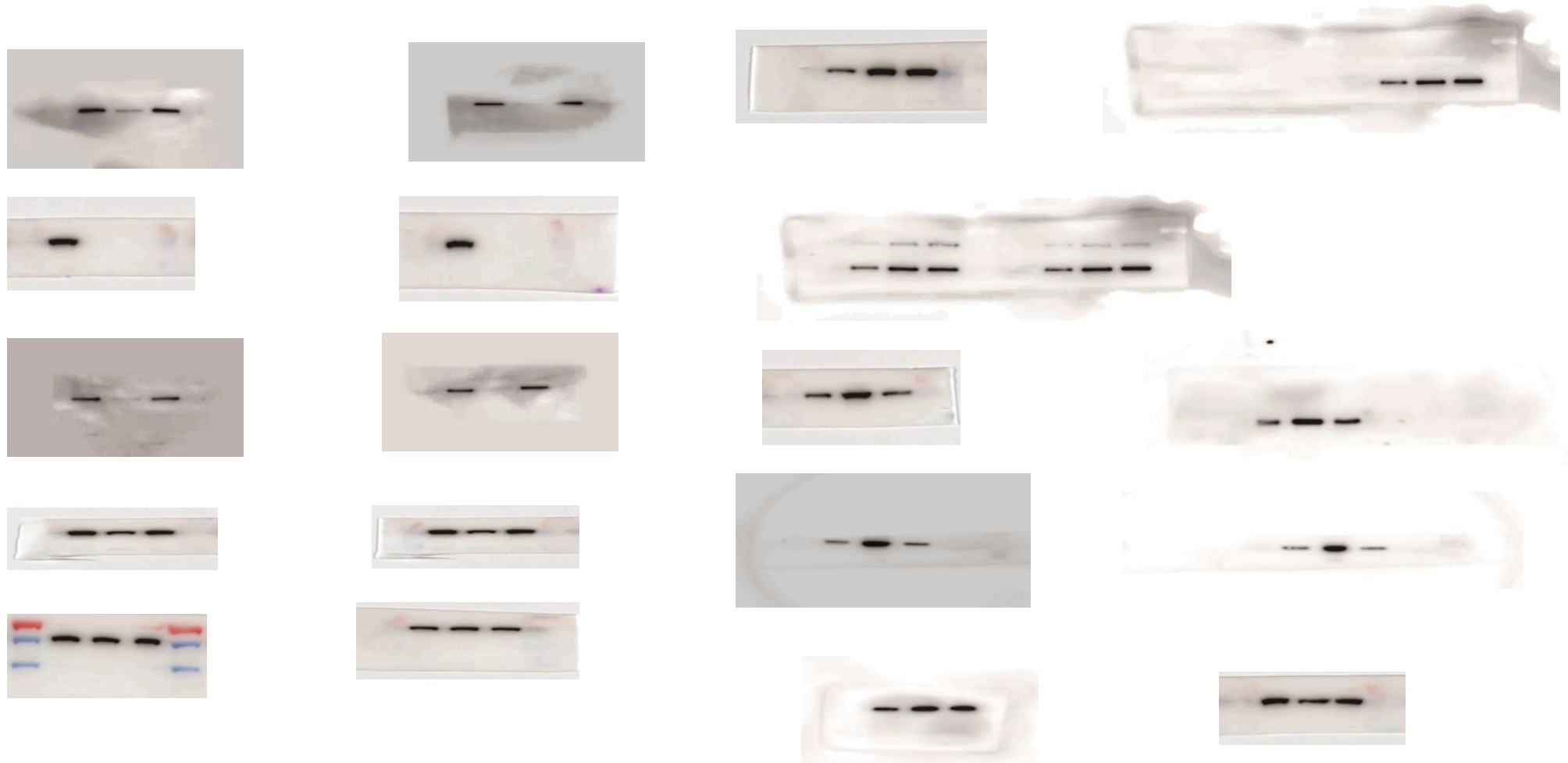

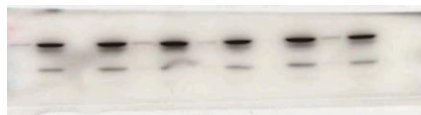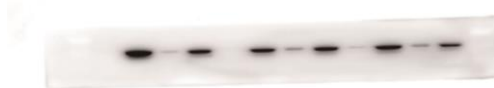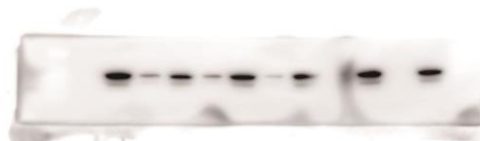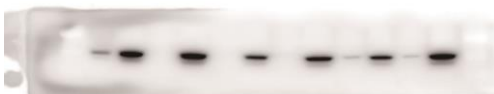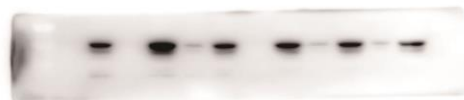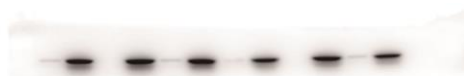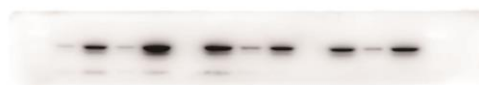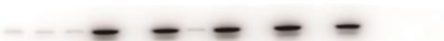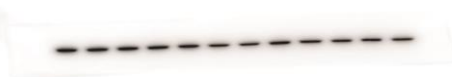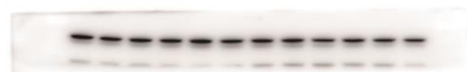

Supplement: Supplementary file 3 — Original Data File [file 41420_2023_1540_MOESM3_ESM.pdf]
